# Bioengineered skin constructs based on mesenchymal stromal cells and acellular dermal matrix exposed to inflammatory microenvironment releasing growth factors involved in skin repair

**DOI:** 10.1186/s13287-023-03535-w

**Published:** 2023-10-26

**Authors:** Luz Correa-Araujo, Leonardo Prieto-Abello, Adriana Lara-Bertrand, Martha Medina-Solano, Linda Guerrero, Bernardo Camacho, Ingrid Silva-Cote

**Affiliations:** 1 Tissue Engineering Unit, Instituto Distrital de Ciencia Biotecnología e Innovación en Salud – IDCBIS, Carrera 32 # 12-81, Secretaria Distrital de Salud, Bogotá, Colombia; 2 Tissue Bank, Instituto Distrital de Ciencia Biotecnología e Innovación en Salud – IDCBIS, Bogotá, Colombia

**Keywords:** Scaffold, hADM, hWJ-MSCs, Growth factor, Skin, Wound repair

## Abstract

**Background:**

Skin tissue engineering is a rapidly evolving field of research that effectively combines stem cells and biological scaffolds to replace damaged tissues. Human Wharton’s jelly mesenchymal stromal cells (hWJ-MSCs) are essential to generate tissue constructs, due to their potent immunomodulatory effects and release of paracrine factors for tissue repair. Here, we investigated whether hWJ-MSC grown on human acellular dermal matrix (hADM) scaffolds and exposed to a proinflammatory environment maintain their ability to produce in vitro growth factors involved in skin injury repair and promote in vivo wound healing.

**Methods:**

We developed a novel method involving physicochemical and enzymatic treatment of cadaveric human skin to obtain hADM scaffold. Subsequently, skin bioengineered constructs were generated by seeding hWJ-MSCs on the hADM scaffold (construct 1) and coating it with human platelet lysate clot (hPL) (construct 2). Either construct 1 or 2 were then incubated with proinflammatory cytokines (IL-1α, IL-1β, IL-6, TNF-α) for 12, 24, 48, 72 and 96 h. Supernatants from treated and untreated constructs and hWJ-MSCs on tissue culture plate (TCP) were collected, and concentration of the following growth factors, bFGF, EGF, HGF, PDGF, VEGF and Angiopoietin-I, was determined by immunoassay. We also asked whether hWJ-MSCs in the construct 1 have potential toward epithelial differentiation after being cultured in an epithelial induction stimulus using an air–liquid system. Immunostaining was used to analyze the synthesis of epithelial markers such as filaggrin, involucrin, plakoglobin and the mesenchymal marker vimentin. Finally, we evaluated the in vivo potential of hADM and construct 1 in a porcine full-thickness excisional wound model.

**Results:**

We obtained and characterized the hADM and confirmed the viability of hWJ-MSCs on the scaffold. In both constructs without proinflammatory treatment, we reported high bFGF production. In contrast, the levels of other growth factors were similar to the control (hWJ-MSC/TCP) with or without proinflammatory treatment. Except for PDGF in the stimulated group. These results indicated that the hADM scaffold maintained or enhanced the production of these bioactive molecules by hWJ-MSCs. On the other hand, increased expression of filaggrin, involucrin, and plakoglobin and decreased expression of vimentin were observed in constructs cultured in an air–liquid system. In vivo experiments demonstrated the potential of both hADM and hADM/hWJ-MSCs constructs to repair skin wounds with the formation of stratified epithelium, basement membrane and dermal papillae, improving the appearance of the repaired tissue.

**Conclusions:**

hADM is viable to fabricate a tissue construct with hWJ-MSCs able to promote the in vitro synthesis of growth factors and differentiation of these cells toward epithelial lineage, as well as, promote in a full-thickness skin injury the new tissue formation. These results indicate that hADM 3D architecture and its natural composition improved or maintained the cell function supporting the potential therapeutic use of this matrix or the construct for wound repair and providing an effective tissue engineering strategy for skin repair.

**Supplementary Information:**

The online version contains supplementary material available at 10.1186/s13287-023-03535-w.

## Background

Skin wounds such as burns and ulcers are complex medical condition requiring lifelong rehabilitation [1]. These injuries are often associated with significant morbidity, impairment of emotional well-being, and decreased patient quality of life [2]. Although numerous advances have been made in skin injury treatment, success is limited due to the reduced functionality of repaired tissue [3], highlighting the urgent need for a safe and transplantable skin alternative. Tissue engineering strategies have been focused on fabricating bioengineered skin constructs that combine cells, scaffolds, and bioactive molecules that promote repair processes [4].

Under normal physiological conditions, skin tissue injury immediately triggers acute inflammation [5]. It has long been thought that the inflammatory response is necessary to provide the growth factors and cytokine signals that orchestrate the cell movements required for tissue repair [6]. Several cytokines, such as interleukins (IL-1α, IL-1β, and IL-6) and tumor necrosis factor-alpha (TNF-α), are critical for the proinflammatory response because one or more of them are capable of regulating the immune microenvironment and stimulating the repair effect of endothelial cells, fibroblasts, and tissue progenitor cells in wounds [7]. This complex physiological response has been highlighted using cell therapy in tissue repair to modulate the inflammatory response.

Mesenchymal stem cells (MSCs) have immunomodulatory and anti-inflammatory properties, allowing their allogeneic use in potential cell therapy approaches for inflammatory or autoimmune diseases [8, 9]. They also produce paracrine factors that recruit other cells to promote tissue repair. As such, they are being used as a promising new therapy for wound healing [10]. Currently, numerous clinical trials based on MSCs therapy for reducing the time of healing of human skin wounds and treating diabetic foot ulcers have been reported [11–15], confirming their therapeutic potential. These cells have been incorporated into different types of scaffolds, such as silk fibroin [16], poly(ɛ-caprolactone)/collagen [17], or chitosan [18], for skin regeneration. So far, very little is known about how the inflammatory microenvironment may affect skin tissue-engineered constructs. In this regard, some studies have shown that the secretion of cytokines and growth factors by MSCs is essential for the processes of re-epithelialization and induction of angiogenesis during wound healing [19]. Taking into consideration subtle differences between MSCs, human Wharton’s jelly mesenchymal stem cells (hWJ-MSCs) may be a more suitable candidate for tissue engineering applications as a result of a high potential to differentiate; they are immune-privileged and are easy to collect [20]. Several studies have shown that paracrine factors derived from hWJ-MSCs promote wound healing by regulating inflammatory responses [21–23], accelerating angiogenesis, increasing migration and proliferation of keratinocytes and fibroblasts, and activating collagen and elastin synthesis by fibroblasts, and promoting regeneration of skin with typical architecture and function [24]. These properties make it a suitable alternative for wound repair [25].

Delivery of these cells into wounds by direct injection has been associated with low viability, transient retention, and overall poor efficacy [26]. In contrast, cells growing on scaffolds may create a controlled microenvironment that may protect them from harmful stimuli such as a wound inflammatory environment. Acellular dermal matrix (ADM) is a biological scaffold that provides skin-native tissue biochemical properties and ultrastructural architecture to support tissue repair [27]. In a preclinical context, ADM has been used to treat chronic skin wounds because they provide molecules that improve intercellular communication and neovascularization in wound surface repair [28]. Although the methods for obtaining ADM are based on the removal of cells from the native tissue, few of them effectively reduce the residual DNA. Moreover, these techniques use substances that can affect the structure of the extracellular matrix and promote cytotoxicity and require long processing times.

Wound healing is a dynamic process involving interactions between cells, extracellular matrix, and growth factors that reconstitute tissue after injury [29]. In the skin, the extracellular matrix (ECM) plays an essential role not only as an architectural support, providing support, tensile strength, and attachment sites for cell surface receptors, but also as a specialized microenvironment for regulating cellular activities and development, including cell proliferation, differentiation, and survival [30]. The ECM also contains bioactive molecules, including growth factors and cytokines that promote wound repair [31].

In this study, we developed a new method to obtain hADM from human cadaveric skin, preserving its ECM proteins such as collagen and elastin with low DNA content. Using hADM, we generated two constructs, first by seeding hWJ-MSCs on hADM (construct 1), and second by creating a human platelet lysate—hPL layer on top of hWJ-MSCs attached to hADM (construct 2). We asked whether an injury-like inflammatory microenvironment created by the addition of IL-1α, IL-1β, IL-6, and TNF-α would affect the synthesis of growth factors involved in skin tissue repair. In addition, given the high differentiation potential of MSCs, we evaluated the ability of hWJ-MSCs in construct 1 to differentiate into epithelial cells by exposing them to an air–liquid system that attempts to mimic the natural skin microenvironment. Moreover, the efficacy of hADM and construct 1 in promoting skin repair was evaluated in full-thickness excisional wounds. Our study found that the in vitro synthesis of wound repair-associated growth factors was maintained in hWJ-MSCs grown on hADM scaffold, even when exposed to a proinflammatory microenvironment. Furthermore, hWJ-MSCs on the dermal matrix can differentiate into an epithelial-like lineage. The in vivo tests showed that the hADM and hADM/hWJ-MSC-based constructs are effective in healing cutaneous lesions. This offers a new approach to skin tissue engineering.

## Materials and methods

### hADM scaffold obtention

Acellular dermal scaffolds were obtained from human cadaveric skin at the Tissue Bank of the Instituto Distrital de Ciencia, Biotecnología e Innovación en Salud (IDCBIS), Bogotá-Colombia. Skin tissue sections used here were remnants from the cutting and regularization process. Each tissue was tested for human immunodeficiency virus (HIV), hepatitis C virus (HCV), hepatitis B virus (HBV), human T-lymphotropic virus (HTLV), Chagas disease, and syphilis positivity. Tissue samples were stored in 85% glycerol, washed twice in 1 × phosphate-buffered saline (PBS) (Gibco, Life Technologies, Carlsbad, CA, USA), and stored in sterile bottles. Each skin sample was subjected to a decellularization process (patent filed in Colombia, No. NC2022/0005963) that included physical, chemical, and enzymatic steps. Briefly, samples were subjected to freeze–thaw cycles, hypertonic solution using 0.5 M and 1 M NaCl (Sigma-Aldrich, St. Louis, MO, USA), enzymatic treatment based on 0,25% Trypsin–EDTA (Gibco, Life Technologies, Canada) for 1 h with constant stirring at 37 °C. Subsequently, hADM was treated with 1% Triton X-100 (Bio-Rad Laboratories, USA) for 24 h and recombinant DNase I 10000U (Roche Diagnostics, Mannheim, Germany) for 6 h; finally, the samples were stored in sterile deionized water at 4 °C.

### Histological analysis of hADM and skin

Histological analysis was performed on hADM and skin samples to evaluate the decellularization process effectiveness. The samples were fixed in 4% paraformaldehyde (PFA) (Panreac, ITW Companies, Darmstadt, Germany), embedded in paraffin, and sectioned (5-μm thickness). To analyze the tissue structure, deparaffinized tissue sections were subjected to hematoxylin and eosin (H&E), Masson’s trichrome, and Verhoeff-van Gieson staining. Each slide was analyzed under a light microscope (Leica, Germany). To detect cell nuclei, samples were stained with 4′,6-diamidino-2-phenylindole (DAPI) (Thermo Fisher Scientific, Waltham, MA, USA); deparaffinized sections were washed in 1 × PBS and exposed to 0.1 µg/mL DAPI. Samples were incubated in the dark for 10 min, repeatedly washed in 1 × PBS, and finally observed by using a fluorescence microscope (Leica DMi8, Germany).

### DNA content assay

To compare the DNA content previous and after of the decellularization process, 10 mg of skin and hADM samples were digested with 20 mg/mL Proteinase K (Thermo Fisher Scientific, Waltham, MA, USA) at 56 °C overnight or until no visible material was observed. DNA was extracted using a Genomic DNA Purification System kit (Promega, Madison, WI, USA). DNA quantification was performed using a NanoDrop-1000 instrument (Thermo Scientific NanoDrop™ 2000/2000c). In this assay, skin from 8 donors was decellularized (*n* = 8). Data were analyzed using GraphPad Prism version 6.0 software, and *p* value < 0.05 was considered statistically significant.

### Tensile testing

The Young’s modulus of skin and hADM samples were measured in an Instron Universal Testing Machine with 0.5 kN load cell. The calibrated length was 21.92 mm, and the tensile speed was 1 mm/min. The laboratory presented temperature conditions of 21.88 °C and relative humidity of 47.76% during the testing. The samples were randomly cut from skin or hADM in the form of 30 × 22 mm rectangular specimens with thicknesses of 0.39 mm. The slope of the elastic zone in the stress–strain graph determined Young’s modulus values. Data were obtained from three skin and hADM donors (*n* = 3). The statistical analysis was made using the software R.

### hWJ-MSCs adhesion and proliferation on hADM scaffold

hWJ-MSCs were previously isolated and characterized, according to Silva-Cote et al. [32]. Human umbilical cords were required to obtain these cells. Participants provided written informed consent to participate in this study, which was reviewed and approved by the Comité de Investigación y Etica, Secretaría Distrital de Salud, Bogotá, Colombia. The title of the approved study was “Design, fabrication and evaluation of constructs generated from biological or synthetic scaffolds and umbilical cord mesenchymal stromal cells for repair of bone, epithelial and cartilage tissue lesions.” To evaluate hWJ-MSCs adhesion on the hADM scaffold, 5 × 10^4^ cells provided by the Advanced Therapies Unit of IDCBIS were seeded on 1 cm^2^ of hADM in DMEM low glucose (Gibco, Life Technologies, Grand Island, NY, USA) supplemented with 10% human platelet lysate (hPL) plus 1% antibiotics, and 160 U heparin (Blau Farmacéutica, Colombia) and cultured for 8 h under standard culture conditions (37 °C, 5% CO_2_). In this study, we used three donors of hWJ-MSCs passage 5. Scanning electron microscopy (SEM) and H&E staining were performed to observe cell adhesion on hADM scaffold.

### Evaluation by SEM

Samples from hWJ-MSCs seeded on the hADM were washed with 1 × PBS (three washes for 3 min), fixed in 4% PFA for 1 h, followed by two washes with deionized water. hADM and skin were exposed to gradient dehydration with ethanol at 30%, 50%, 70%, 90%, and 96% for 15 min and 100%, three times for 10 min each. The critical point drying was conducted using CPD020 Balzers Union and 1,1,3,3,3-Hexamethyldisilazane (HMDS) (Sigma-Aldrich, St. Louis, MO, USA) in three washes of 10 min each. Subsequently, a gold coating under vacuum was performed using a sputtering machine (Quorum Q150 RES) at 1 kV and 5 mA for 60 s. The gold-coated samples were imaged by SEM (ZEISS EvoMA10, Germany) at an accelerating voltage of 20 kV. Magnification of 1000× was used, and images were processed using the software ImageJ.

### Cell viability and proliferation assay

In order to evaluate whether the hWJ-MSCs were viable on the scaffold, a live/dead assay (Invitrogen, Waltham, MA, USA) was performed. Briefly, 5 × 10^4^ cells were seeded and maintained for 24 h on 1 cm^2^ of hADM scaffold and tissue culture plate (TCP) surface. The culture medium was then removed, and the cells were washed with 1 × PBS. 200 μL Live/dead solution (2 μM green-fluorescent calcein-AM and 4 μM red-fluorescent ethidium homodimer-1) was added to each well and incubated at 37 °C for 20 min. The wells were gently washed with 1 × PBS, and the labeled cells were immediately observed under a fluorescence microscope (Leica DMi8, Germany).

On the other hand, a resazurin assay (Sigma-Aldrich, St. Louis, MO, USA) was performed to analyze hWJ-MSCs proliferation on the hADM scaffold. Briefly, 5 × 10^4^ hWJ-MSCs were seeded on hADM scaffold and cultured in hPL-supplemented DMEM. The culture medium was removed, and the cells were washed with 1 × PBS. Each well was exposed to 500 µL 1:100 resazurin in culture medium for 4 h at standard culture conditions. Finally, 100 μL of supernatant was analyzed at 570 nm excitation and 600 nm emission using a Synergy HTX plate reader (BioTek, USA). These assays were performed for 5 days. Data were analyzed according manufacturer indications. The following formula was used to determine the percentage of cell proliferation on the hADM scaffold.

$$\% \, \;{\text{Cell}}\;{\text{ proliferation}} = \frac{{\left( {\varepsilon_{{{\text{OX}}}} } \right)\lambda_{2} A\lambda_{1} {-}\left( {\varepsilon_{{{\text{OX}}}} } \right)\lambda_{1} A\lambda_{2} }}{{(\varepsilon_{{{\text{OX}}}} )\lambda_{2} A^\circ \lambda_{1 \, - } (\varepsilon_{{{\text{OX}}}} )\lambda_{1} A^\circ \lambda_{2} }} \times 100$$

where (*ε*_OX_) *λ*_2_ = Molar Extinction Coefficient for alamarBlue^®^ at 600, *Aλ*_1_ = Absorbance hWJ-MSCs seeded on dermal scaffold (test well) at 570 nm, (*ε*_OX_) *λ*_1_ = Molar Extinction Coefficient for alamarBlue^®^ at 570 nm, *Aλ*_2_ = Absorbance hWJ-MSCs seeded on dermal scaffold (test well) at 600 nm, and *A*°*λ*_1_ = Absorbance for positive control well at 570 nm, *A*°*λ*_2_ = Absorbance for positive control well at 600 nm.

According to this formula, the difference (percentage) of resazurin reduction between hWJ-MSCs seeded on hADM scaffold and TCP surface used as positive control reflects the growth percentage of hWJ-MSCs seeded on the dermal matrix. Data are presented as mean ± standard deviation (SD) of samples from three donors (*n* = 3). Data from this evaluation were analyzed using GraphPad Prism version 6.0. *p* values < 0.05 were considered statistically significant.

### Constructs formation based on hADM scaffold and hWJ-MSCs

In the present study, two types of constructs have been evaluated. The first was a hADM scaffold recellularized with hWJ-MSCs (construct 1). The second was the recellularized scaffold covered with a hPL clot (construct 2). Briefly, 20% hPL-supplemented DMEM (without heparin) covered construct 1, which was incubated for 20 min. For both constructs, cells were seeded onto the scaffolds as described above. Constructs 1 and 2, as well as hWJ-MSCs grew on TCP, were treated with a cocktail of proinflammatory cytokines 10 ng/mL IL-1α (570,006, Biolegend, San Diego, CA, USA), IL-1β (579406, Biolegend, San Diego, CA, USA), IL-6 (570806, Biolegend, San Diego, CA, USA), and TNF-α (570106, Biolegend, San Diego, CA, USA). The supernatant was collected at 12, 24, 48, 72, and 96 h after the inflammatory stimulus. It should be noted that the cytokines were refreshed at 48 h. Cells grown on TCP, construct 1, and construct 2 without proinflammatory treatment were evaluated as controls.

### Detection of skin lesion repair growth factors from constructs

Skin repair growth factors’ concentration was measured using a custom magnetic microbead-based immunoassay (R&D Systems, Minneapolis, USA). The assay-specific data sheet reports less than 0.5% cross-reactivity and interference. Data from the immunoassay were analyzed by a Luminex 200 instrument (Texas, USA). The supernatants from cells grown on TCP, construct 1, and construct 2 stimulated and non-stimulated were collected at 12, 24, 48, 72, and 96 h. To detect angiogenic growth factors, we evaluated the levels of platelet-derived growth factor (PDGF), vascular endothelial growth factor (VEGF), and angiopoietin 1 (Ang1). We also analyzed factors associated with epithelialization, such as hepatocyte growth factor (HGF), epidermal growth factor (EGF), and basic fibroblast growth factor (bFGF). Similarly, the concentration of growth factors in hPL-supplemented DMEM was also analyzed during the immunoassay. The procedure was performed according to the manufacturer’s instructions, and the concentration of each protein was expressed in pg/mL. Data were presented as mean ± SD/error standard of samples from three donors (*n* = 3). Data from this evaluation were evaluated using a three-way ANOVA test with GraphPad Prism version 6.0. *p* values < 0.05 were considered statistically significant.

## Epithelial differentiation of hWJ-MSCs on hADM scaffold

To assess the ability of hWJ-MSCs seeded on the hADM scaffold to differentiate toward the epithelial lineage, construct 1 was maintained in keratinocyte growth medium (KGM) (SingleQuots: CC-4131, Lonza, Germany) or hPL-supplemented DMEM, in both conditions the constructs were seeded on transwell inserts (air–liquid system) for 14 days under standard culture conditions. Medium was changed every three days. Cells grown on TCP and cultured in hPL supplemented DMEM were used as controls. For immunocytochemical analysis, samples were fixed with 4% PFA.

### Immunocytochemistry

Epithelial and mesenchymal markers were used to evaluate the differentiation of cells grown on hADM. Briefly, samples were washed three times with 1 × PBS, then permeabilized with 0.5% Triton X-100 for 10 min, washed twice with 1 × PBS and 0.5% PBS-BSA (bovine serum albumin), blocked with 5% PBS-SFB (fetal bovine serum) for 1 h. The samples were incubated overnight at 4 °C with the following antibodies: 2 µg/mL anti-filaggrin (NBP1-87528, Novus Biologicals, Centennial, CO, USA), 10 µg/mL anti-plakoglobin (H00003728, Abnova, Cambridge, UK), 5 µg/mL anti-involucrin (924401, Biolegend, San Diego, CA, USA), and 2.5 µg/mL anti-vimentin (677804, Biolegend, San Diego, CA, USA) with constant stirring. The primary antibody was then removed, and the samples were washed five times with 1 × PBS. Each sample was incubated with the corresponding secondary antibody conjugated with Alexa Fluor 594: 1:1000 Goat anti-rabbit (ab150080, Abcam, Cambridge, UK) or 1:1000 Goat anti-mouse (ab150116, Abcam, Cambridge, UK), diluted in 0.5% PBS-BSA for 1 h at 4 °C. Specific information about each antibody is described in Additional file 1: Table S1. Five washes with 1 × PBS were applied to remove each antibody. Additionally, 0.1 µg/mL DAPI was used for counterstaining, and the wash cycle was repeated. The samples were kept in deionized water and observed using a fluorescence microscope (Leica DMi8, Germany).

### In vivo repair potential of construct and hADM

The animal protocol was approved by the Institutional Animal Care and Use Committee of ANESTCOL S.A., Bogota, Colombia (Protocol Codig:094). Six female Yorkshire pigs (10 weeks old and weighing approximately 45 kg) were used to evaluate the wound healing capacity of construct 1 and hADM. All animals were humanely treated and maintained in an acclimation period. A nose cone attached to the anesthesia machine was used to deliver isoflurane (3–4%) during the procedure. A 0.9% NaCl solution was administered intravenously into an ear vein to maintain hydration. Animals were operated on in the prone position, and the dorsal region was shaved and then washed with Chlorhexidine^®^. The wound area was demarcated with a sterile acetate frame, and three full-thickness surgical wounds, 3 cm × 3 cm, were created at the level of the panniculus adiposus, representing the removal of all layers of skin. Each wound was covered with the corresponding Construct 1 or hADM scaffold, and the untreated wound served as the negative control. Construct 1 or hADM scaffold was sutured through the wound margin and the center of the lesion. Each wound was then covered with sterile gauze, Fixomull Stretch, and a dressing for 8 days, as is typical in the clinical management of injuries. After these elements were removed, the wounds were monitored until day 30. Photographs of the wounds were taken with a film transparency ruler at 0, 8, and 30 days, and the contour size was analyzed using ImageJ software, and the wound closure rate was calculated according to the following formula:

$${\text{Wound}}\;{\text{ Closure}}\; \left( \% \right)\; = \;\frac{{{\text{Area}}\;{\text{ of}}\;{\text{ original}}\;{\text{ wound}} - {\text{Area}}\;{\text{ of}}\; {\text{actual}}\;{\text{ wound}}}}{{{\text{Area}}\;{\text{ of}}\;{\text{ original}} \;{\text{wound}}}}\;*\;100\%$$

### Histological evaluation

After 30 days, the euthanasia protocol was performed using Euthanex and tissue sections from the repaired zone (treated with construct 1 or hADM); untreated wounds and healthy skin were fixed with 4% PFA, embedded in paraffin, and sectioned at 4 μm. After deparaffinization, hematoxylin and eosin (HE) and Masson’s trichrome staining were performed. Images were captured with a light microscope (Leica DMi8, Germany). The following parameters were used to evaluate wound re-epithelialization and collagen fiber thickness in the wound healing process: −: none, +: mild, ++: moderate, +++: marked.

### Statistical analysis

Experiments were performed in triplicate, and values were expressed as mean ± SD/standard error of mean. Comparisons between different groups were assessed by three-way analysis of variance (ANOVA). Two-way ANOVA and Tukey’s test were used to analyze the wound closure data. *p < 0.05, **p < 0.01, ***p < 0.001, and ****p < 0.0001 were considered statistically significant, ****p < 0.0001. GraphPad Prism version 6.0 was used for data treatment, and images were processed with ImageJ software.

## Results

### Biological scaffold for tissue engineering

**Fig. 1:**
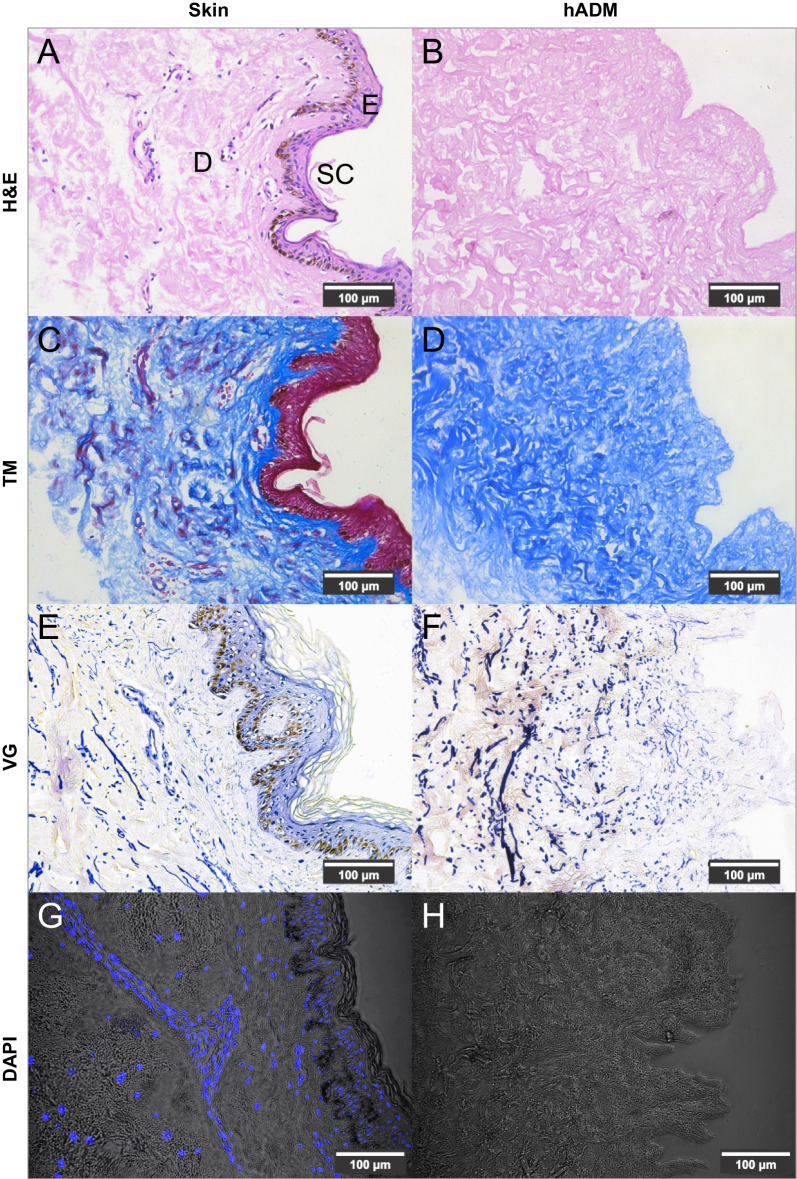
Histological characterization of skin and hADM. Skin structure by Hematoxylin and Eosin, and Masson’s trichrome staining (**A**, **C**): Epidermis (*E*), dermis (*D*), and stratum corneum (*SC*) are observed. For hADM, the extracellular matrix (pink) and collagen fibers (blue) are identified (**B**, **D**). Elastic fibers (dark blue) in skin (**E**) and hADM (**F**) are observed by Verhoeff-Van Gieson staining. Scale bar: 100 μm. Detection of cellular nuclei in skin (**G**) and absence of those in hADM (**H**) by DAPI staining. Scale bar: 100 μm. n = 8. hADM: Human acellular dermal matrix. H&E: Hematoxylin and Eosin. *TM* Masson’s trichrome, *VG* Verhoeff-Van Gieson

After decellularization, quality parameters such as absence of cells and residual DNA and preservation of native extracellular matrix were evaluated, which are essential criteria for characterization of a biological scaffold [33]. Hematoxylin and eosin (H&E) staining of skin revealed stratified epithelium, melanocytes and cell nuclei (Fig. 1A). These elements were not observed in hADM. This result was observed in all evaluated donors (Fig. 1B). Skin Masson’s trichrome staining confirmed the presence of skin layers and dermal papillae (Fig. 1C).

For hADM, a cell-free scaffold with extracellular matrix containing preserved collagen structure (stained blue) and dermal papillae were observed (Fig. 1D). Similarly, the elastic fibers were not affected by decellularization process (Fig. 1E, F). Cell nuclei (DAPI staining) were observed in skin (Fig. 1G) but not in hADM (Fig. 1H). These findings suggest that the treatment eliminated human skin cells.

**Fig. 2:**
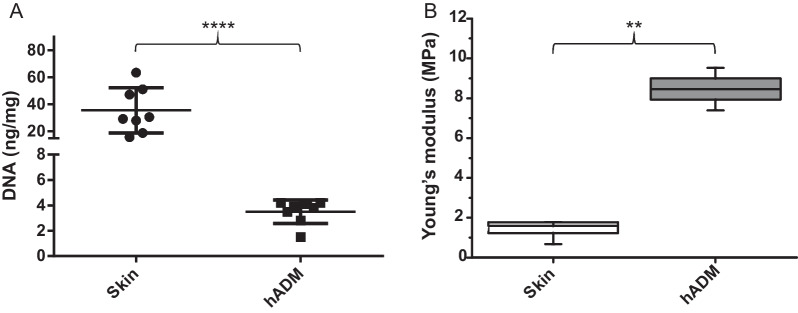
Biomechanical characterization of skin and hADM. Reduction in DNA concentration after the decellularization process to obtain hADM compared to native skin (**A**). *n* = 8, *p* < 0.0001 (****). Mechanical properties of skin and hADM sections (**B**) represented by Young’s modulus (MPa). *n* = 3, *p* < 0.01 (**). *hADM* human acellular dermal matrix

DNA content is one of the most critical parameters for characterizing decellularized biological scaffolds. A concentration of 4 ng/mg or less indicates that DNA residues have been removed from the tissue [34]. As we expected, DNA content in hADM was significantly lower with respect to skin (*P* < 0.0001). The average DNA content of skin was 33.5 ng/mg, whereas the DNA content of decellularized dermal matrix was 3.83 ng/mg (Fig. 2A). On the other hand, a mechanical analysis was evaluated by examining the Young’s modulus of skin and hADM, as shown in Fig. 2B. The value of this property was 1.41 ± 0.64 MPa and 8.46 ± 1.51 MPa for skin and hADM, respectively.

### hWJ-MSCs growing on biological scaffold

**Fig. 3:**
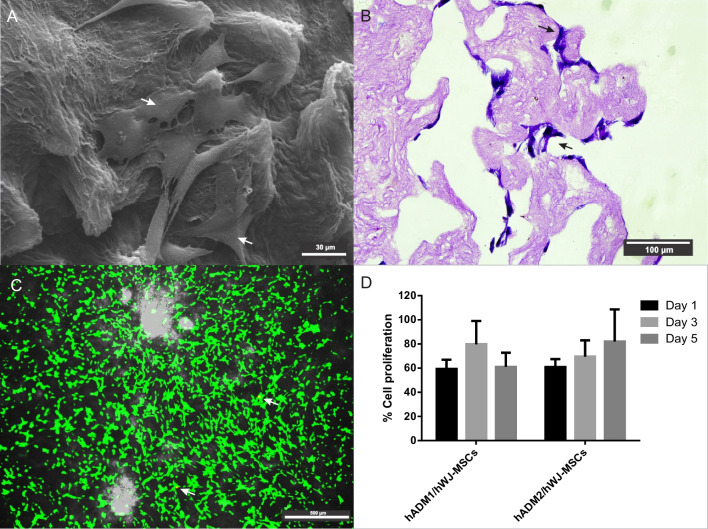
hADM biocompatibility and construct formation. SEM shown the hWJ-MSCs attachment on hADM, scale bar = 30 μm (**A**), and Hematoxylin and Eosin staining (**B**) shown the infiltration of hWJ-MSCs inside hADM scaffold, scale bar = 100 μm, as indicated by arrows, respectively. In recellularized hADM, the viability of hWJ-MSCs (green) was more than 98% after 24 h, scale bar = 100 μm (**C**). Resazurin-based assay to monitor the proliferation of hWJ-MSCs on hADM (two donors: hADM1 and hADM2) during the culture process (D), values were normalized using a control group (cells grown on TCP). *n* = 3, *p* < 0.05. *TCP* tissue culture plate

Cells were seeded on the biological scaffold to demonstrate the growth of hWJ-MSCs on the hADM surface. After 24 h, cell adhesion and cell spreading on the scaffold surface and dermal papillae were observed under an electronic microscope (Fig. 3A). We also evaluated cell adhesion on day 14 after culture. At this time, hWJ-MSCs were observed on the surface and infiltrated inside of hADM (Fig. 3B).

Cell viability on the dermal scaffold was greater than 98% after 24 h of culture as shown in Fig. 3C. The ability to promote cell proliferation is an essential criterion for the quality of a biological scaffold. Therefore, we evaluated the proliferation of hWJ-MSCs on the hADM scaffold. For both hADM donors, cell growth was greater than 70% compared to hWJ-MSCs on TCP at day 3 after culture (normalized data in the graph) (Fig. 3D). According to these results, hADM promoted cell proliferation.

### Tissue engineering constructs produce epithelialization growth factors

**Fig. 4:**
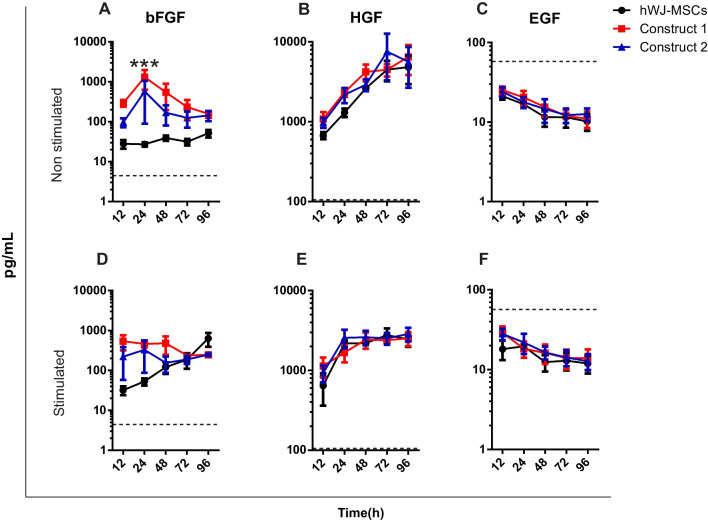
Release of growth factors associated with epithelization without proinflammatory cytokines, bFGF (**A**), HGF (**B**), EGF (**C**) and treated with proinflammatory cytokines, bFGF (**D**), HGF (**E**), and EGF (**F**). Dashed lines reflect the concentration of factors in hPL-supplemented DMEM. *n* = 3; values are presented on a logarithmic scale. *p* < 0.05(*), *p* < 0.001(***)

At this point, we proceeded with seeding hWJ-MSCs on hADM, considered as construct 1, and construct 2 was hWJ-MSCs on hADM covered with a hPL clot. All conditions were then exposed to a called “inflammatory microenvironment” containing IL-1α, IL-1β, IL-6, and TNF-α. After incubation with these cytokines, we quantified bFGF, HGF, EGF, Ang I, VEGF, and PDGF, all together fundamental for skin wound repairing process such as granulation tissue formation, angiogenesis, cell proliferation, epithelialization, and extracellular matrix production [35]. Regarding epithelialization growth factors, low levels of bFGF were reported in hPL-supplemented DMEM (control), whereas a significant production was reported in construct 1 without proinflammatory stimulation at 24-h post-culture. This value was statistically significant compared to hWJ-MSCs (*p* value < 0.05) (Fig. 4A). For constructs exposed to cytokine cocktail, a higher production was observed between 12- and 48-h post-treatment compared to hWJ-MSCs (Fig. 4D). However, no statistical differences were observed.

Increased HGF levels were observed in both constructs and hWJ-MSCs without stimulation throughout the assay (Fig. 4B). In hWJ-MSCs and cytokine-stimulated constructs, HGF production increased during the first 24 h, after which the levels remained constant (Fig. 4E). It should be noted that HGF secreted in untreated construct 2 was highly significant compared to that exposed to a proinflammatory microenvironment at 72 h (*p* < 0.05). The same result was observed for construct 1 at 96 h (*p* < 0.05). In addition, growth factor levels in the study groups were significantly higher (672–7548 pg/mL) than those found in the culture medium, which averaged 4 pg/mL. Based on these results, lower production of both bFGF and HGF was reported in constructs and hWJ-MSCs exposed to inflammatory cytokines compared to unstimulated groups. On the other hand, as shown in Fig. 4C, F, EGF levels were detected in the culture medium, and its levels decreased in all constructs and hWJ-MSCs during the study period. This result was observed in both control and stimulated groups.

### Angiogenic factors synthesis is not affected by hADM

**Fig. 5:**
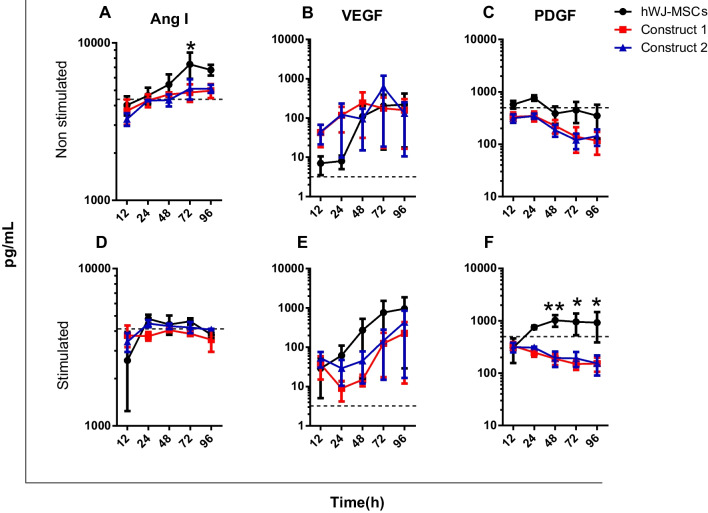
Release of growth factors associated with epithelization without proinflammatory cytokines, Ang I (**A**), VEGF (**B**), PDGF (**C**) and treated with proinflammatory cytokines, Ang I (**D**), VEGF (**E**), and PDGF (**F**). Dashed lines reflect the concentration of factors in hPL-supplemented DMEM. *n* = 3; values are presented in logarithmic scale. *p* < 0.05(*), *p* < 0.01(**)

Growth factors production involved in angiogenesis is essential during the skin repair process. A progressive increase in Ang I was observed in constructs and hWJ-MSCs without proinflammatory stimulation, especially in hWJ-MSCs at 72 h, whose production was statistically higher compared to construct 1 (*p* < 0.05) (Fig. 5A). Regarding the treated groups, Ang I levels in the constructs and cells were low or equal to those found in the culture medium, indicating that hADM did not affect Ang I synthesis (Fig. 5D). For VEGF, a higher production was observed in constructs compared to hWJ-MSCs without cytokine treatment until 24 h; after this time, we observed a similar production of VEGF in both constructs and hWJ-MSCs with and without proinflammatory stimulus (Fig. 5E, B). These levels were higher than those found in the culture medium, and no statistically significant differences were observed between the groups analyzed.

Another essential growth factor for angiogenesis is PDGF. According to the results, a higher concentration was detected in the culture medium (500 pg/mL) with respect to treated and untreated constructs. Over time, PDGF synthesis decreased in constructs independent of proinflammatory stimulus (Fig. 5C, F), whereas in cells seeded on TCP and stimulated with proinflammatory cytokines, the factor production was highly significant from 48- to 96-h post-treatment (*p* < 0.01 and *p* < 0.05, respectively). No significant differences were reported for the non-stimulated groups (hWJ-MSCs and constructs). Altogether, we confirmed that hADM did not affect the production of angiogenic factors by constructs.

### hADM scaffold promotes the hWJ-MSCs epithelial differentiation

**Fig. 6:**
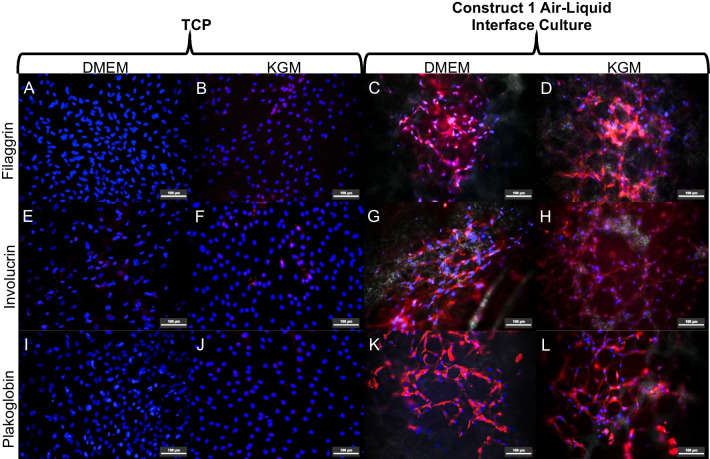
hWJ-MSCs grown on hADM scaffold could differentiate into epithelial-like cells. Absence of filaggrin on day 14 in hWJ-MSCs grown on TCP with and without epithelial medium (**A** and **B**). Filaggrin production in hWJ-MSCs seeded on hADM and air–liquid system (**C**) and treated with epithelial induction medium (**D**). Involucrin synthesis in hWJ-MSCs seeded on TCP (**E** and **F**), in the construct with hPL-supplemented DMEM (**G**) and cultured with epithelial induction medium (**H**). Plakoglobin absence in the monolayer culture (I and J), and Plakoglobin synthesis in the construct cultured with hPL-supplemented DMEM or epithelial medium, respectively (**K** and **L**). Scale bar = 100 μm. *n* = 3. *TCP* tissue culture plate, *KGM* keratinocytes growth medium or epithelial induction medium

**Fig. 7:**
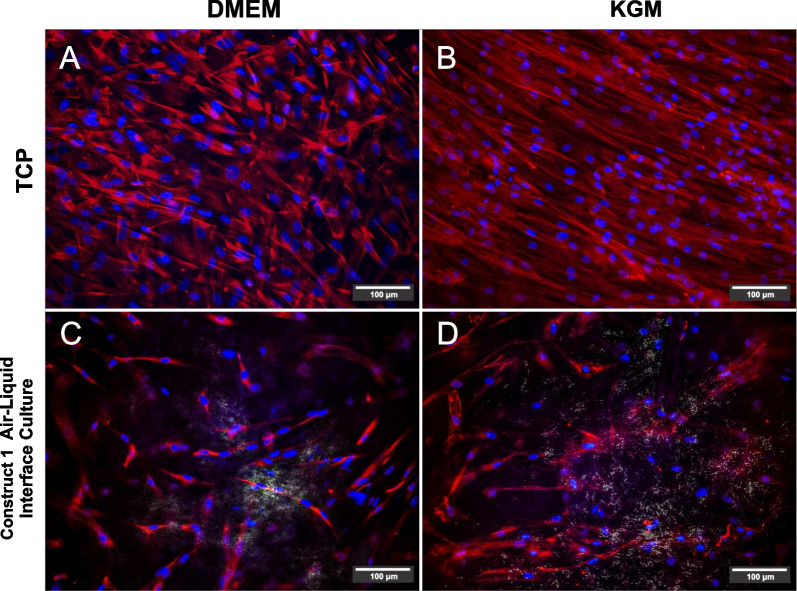
Vimentin expression (mesenchymal marker) by hWJ-MSCs grown on TCP and cultured with hPL-supplemented DMEM (**A**) or epithelial culture (**B**). hWJ-MSCs grown on the scaffold and air–liquid system maintained in hPL-supplemented DMEM or epithelial culture (**C** and **D**, respectively) Scale bar = 100 μm. *n* = 3. *TCP* tissue culture plate, *KGM* keratinocytes growth medium or epithelial induction medium

From this point, we carried out all experiments using construct 1. We maintained the constructs in an air–liquid system with KGM and used constructs cultured in hPL-supplemented DMEM and hWJ-MSCs on TCP as controls to assess whether hWJ-MSCs could differentiate into epithelial-like cells. Expression of epithelial markers, including filaggrin (Fig. 6A, B), involucrin (Fig. 6E, F), and plakoglobin (Fig. 6I, J), was not observed in hWJ-MSCs cultured in DMEM or KGM. In contrast, intracellular synthesis of these markers was observed in construct 1 maintained in hPL-supplemented DMEM (filaggrin: Fig. 6C; involucrin: Fig. 6G; plakoglobin: Fig. 6K) and KGM (Fig. 6D, H, L, respectively). On the other hand, vimentin (mesenchymal marker) expression was observed in hWJ-MSCs seeded on TCP and cultured in hPL-supplemented DMEM or KGM (Fig. 7A, B, respectively). However, we found reduction in this marker in constructs cultured in air–liquid system and hPL-supplemented DMEM or KGM (Fig. 7C, D, respectively).

### Wound healing ability of hADM and hWJ-MSCs/hADM construct

**Fig. 8:**
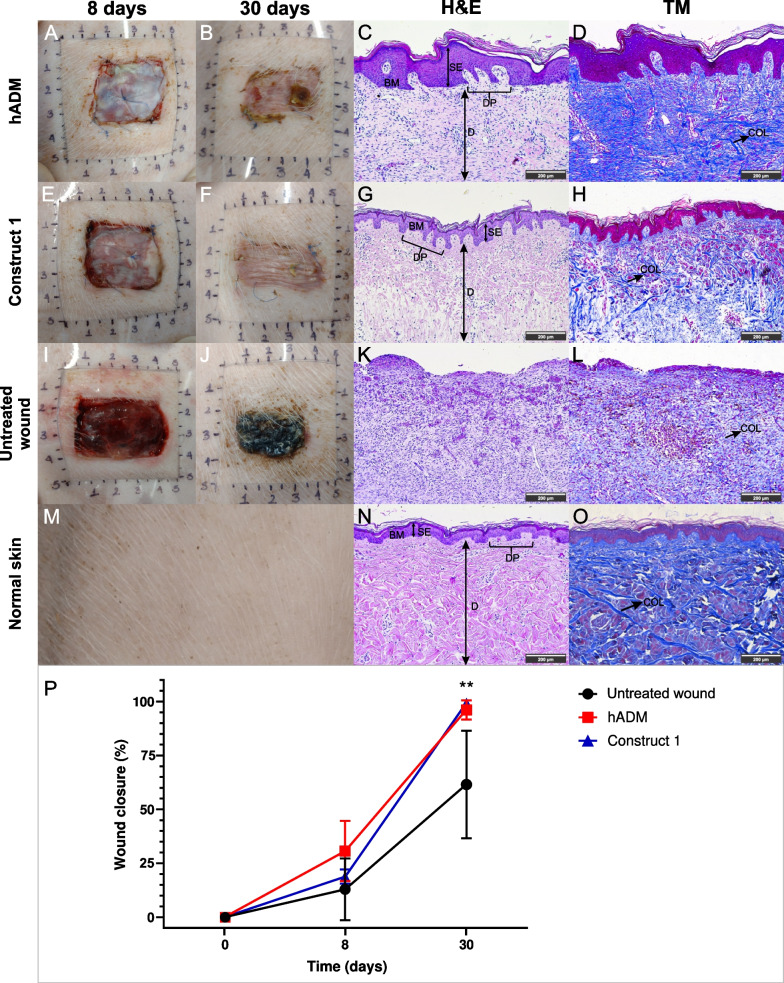
Skin wound repair potential of construct 1 and hADM in an in vivo model. Macroscopic appearance of the wounds at 8 and 30 days after treatment: hADM (**A** and **B**), construct 1 (**E** and **F**), untreated wound (**I** and **J**), normal skin (**M**). Representative images of histological sections of repaired wounds at day 30 using hADM (**C** and **D**), construct 1 (**G** and **H**), untreated wound (**K** and **L**), and normal skin (**N** and **O**). Hematoxylin & eosin (**C**, **G**, **K**, **N**) and Masson trichrome stain (**D**, **H**, **I**, **O**). Wound closure rate (**P**). SE: stratified epithelium, BM: basement membrane, *DP* dermal papillae, *D* Dermis, *COL* collagen fibers. Scale bar = 200 μm. *n* = 6. *p* < 0.01(**)

To evaluate the in vivo wound healing potential of construct 1 and hADM, we used a porcine model (Yorkshire), as porcine wound healing is often used as a model for human wound healing. At 8 days after implantation, both hADM and construct showed no macroscopic signs of inflammation (Fig. 8A, E), while the untreated wound showed inflammation (Fig. 8I). At 30 days, the treatments were favorably engrafted, accompanied by complete wound closure with minimal contraction in construct 1 (Fig. 8F, P) and 95% closure in hADM (Fig. 8B, P). In contrast, the untreated wound showed partial closure (Fig. 8P).

Histologic analysis of skin biopsies showed epithelial restoration and key repair indicators in wounds treated with hADM scaffold and Construct 1 through the formation of basement membrane, dermal papillae, and stratified epithelium where the stratum corneum, lucidum, granulosum, and spinosum were identified (Fig. 8C, G; Table 1); furthermore, the synthesis of thin and thick collagen fibers was observed (Fig. 8D, H; Table 1). Notably, no bleeding or necrosis was observed, demonstrating the biocompatibility of construct 1 and hADM. Histological results showed similarities with healthy skin (Fig. 8M), such as epithelial structure (Fig. 8N) and collagen fiber appearance (Fig. 8O), especially in wounds treated with construct 1. On the contrary, in the untreated defect (negative control), an evident contraction with the formation of necrotic tissue (Fig. 8J) and the presence of an inflammatory infiltrate (Fig. 8K), as well as few collagen fibers (Fig. 8L), suggested a low restoration of damaged tissue.

**Table 1:** Histological evaluation of wounds

	hADM	Construct 1	Untreated wound
*Epithelialization*			
Stratum Basale	+++	+++	−
Stratum Spinosum	+++	+++	−
Stratum Granulosum	+++	+++	−
Stratum Lucidum	+++	+++	−
Stratum Corneum	+++	+++	−
*Collagen fibers*			
Thick fibers	++	+++	−
Thin fibers	+++	++	+++

Histological evaluation for hADM, Construct 1 and Untreated wound. Criteria for estimation of epithelialization and collagen fibers thickness from biopsy samples: (−: none, + : mild, ++ : moderate, +++ : marked). *n* = 6

In vivo testing demonstrated that hADM and construct 1 promote efficient repair of full-thickness skin injury through new tissue formation and significant improvement in the appearance of scarring.

## Discussion

Biological scaffolds are used in skin tissue engineering because these matrices have a high extracellular protein content, which promotes cell growth and differentiation [36]. Acellular dermal matrices have been successfully used in various clinical applications and have been considered as a therapeutic artificial dermal substitute for the treatment of chronic skin wounds [37], with a potential for functional improvement through the addition of specific biological components [28], such as stem cell seeding, because a coordinated synergy between the ECM, cells, and some specific biomolecules promoting tissue repair and regeneration processes. Although these three components are relevant in skin wound healing, the ECM has the critical function of acting as a biological platform where this interaction is established [37].

In this study, we used a novel method to obtain hADM whose acellularity was demonstrated by histological analysis and DNA content. DNA removal is a critical indicator of successful decellularization. DNA concentrations in hADM were less than 4 ng/mg, a concentration that confirmed the acellularity of the biological scaffold according to a previous report on human tissue decellularization [34]. Furthermore, these concentrations were lower than those reported for commercially available hADM (MatrACELL, GraftJacket and Alloderm^®^) [38]. As a consequence, our decellularization process represents a novel alternative to reduce the amount of residual DNA during the obtaining of a cell-free matrix.

In addition, this methodology preserves ECM proteins, which promotes the adhesion and survival of cells seeded on the scaffold. We have identified extracellular proteins such as collagen type I and elastin in hADM. These proteins are the major components of the native structure of the dermis and strongly regulate its strength and elasticity [39]. It was evident that hADM became stiffer after the decellularization process; a similar result was described by Perez et al. [40], who reported Young’s modulus values of skin and hADM of 14.27 ± 8.71 MPa and 19.26 ± 10.35 MPa, respectively. However, Young’s modulus values of hADM obtained in our study were within the range of average skin elasticity (4.6–20 MPa for tensile tests) [41]. Therefore, the decellularization process did not affect the mechanical properties of hADM, suggesting that hADM can be used as a skin dressing without the risk of deformation.

Similarly, due to the high cell viability and proliferation on the hADM scaffold, two tissue constructs were generated to assess whether a proinflammatory microenvironment affects the production of growth factors associated with skin wound repair: hWJ-MSCs/hADM (Construct 1) and Construct 2, which included platelet lysate clot as a nutrient supplement to support hWJ-MSCs/hADM, considering the potential clinical translation of the construct. Both constructs 1 and 2 were exposed to IL1α, IL1β, IL-6, and TNFα, for which we simulated an inflammatory microenvironment typical of a skin lesion [42] since inflammation is known to be one of the earliest stages associated with the injury process and subsequent tissue repair due to inducing secretion of bioactive substances from adjacent non lesioned tissue, and modulating cell behavior [43].

Notably, high production of bFGF was reported in both constructs (approximately 1000 pg/mL), independent of the inflammatory stimulus; these levels were higher than those reported in bone marrow MSCs grown on biosynthetic scaffolds, in which bFGF production did not exceed 15 pg/mL [44]. This growth factor is essential for activating keratinocytes, fibroblasts, and endothelial cells. It is also involved in all phases of wound repair, including angiogenesis, granulation tissue formation, epithelialization, and tissue remodeling [45, 46]. Moreover, the increased production of bFGF in the constructs without proinflammatory stimulus suggests an effect of the hADM scaffold on hWJ-MSCs, which is attributed to the possible influence of the structure or growth factors in the scaffold on the activation of hWJ-MSCs [47]. This factor and VEGF promote angiogenesis by attracting fibroblasts that secrete ECM components necessary to rebuild and remodel injured tissue [48].

Likewise, we observed a progressive increase in HGF in the unstimulated constructs versus the cytokine-treated constructs, indicating that the inflammatory microenvironment affected this factor production in the constructs. HGF levels (672–7548 pg/mL) were higher than those reported by Qazi [44], which ranged from 50 to 130 pg/mL for hWJ-MSCs grown on biosynthetic scaffolds. This result is relevant because HGF is an antifibrotic factor that enhances metalloproteinase activation and inhibits fibroblast differentiation into myofibroblasts [49], promoting scar reduction [50].

Another critical factor is VEGF, an angiogenic factor highly produced in the constructs, especially in those not treated with cytokines. This result suggests that the constructs may improve vascularization [51], which is necessary to provide oxygen and nutrients to the wound bed [52]. Another angiogenic factor is angiopoietin I; its secretion increased progressively in all unstimulated conditions, suggesting that the inflammatory microenvironment affects the production of this factor. This protein interacts with VEGF to promote blood vessel maturation, stabilization, and remodeling [53, 54]. On the other hand, PDGF is also known to be an important mediator of angiogenesis. In this work, we found that this factor decreased in all conditions except when hWJ-MSCs were stimulated, indicating the effect of proinflammatory cytokines on PDGF production. The reduction in PDGF and EGF levels may indicate a massive consumption of these factors by hWJ-MSCs, considering that they are involved in cell proliferation processes [55, 56].

Altogether, these results indicate that hADM promoted the secretion of bFGF and did not affect the production of other growth factors by hWJ-MSCs, even under exposure to an inflammatory microenvironment. Therapeutically, these findings are significant since the functionality of bFGF mainly allows the activation of specialized cells (keratinocytes) to initiate their migration and cell proliferation in the tissue repair process [57]. It should be noted that the release of growth factors in both constructs exposed or not to proinflammatory cytokines was similar. Thus, we decided to evaluate epithelial differentiation patterns and in vivo repair potential using construct 1.

It has been reported that MSCs can differentiate into various cell types, including non-mesodermal cell lineages, such as vascular endothelium [58], neural cells [59], and epithelial lineages [60]. However, they require specialized treatments to induce this differentiation into a specific cell type [33]. Researchers have shown that hWJ-MSCs can differentiate into epithelial-like cells when a specific stimulation is applied [61]. The primary strategy to differentiate hWJ-MSCs into keratinocytes is to establish inductive conditions such as specific culture supplements and to use an air–liquid system. Based on the above, the effect of hADM on differentiating hWJ-MSCs was evaluated in this study.

Interestingly, we found that hADM induced the differentiation of hWJ-MSCs into epithelial-like cells in an air–liquid interface cell culture system that mimicked the microenvironment of in vivo epithelial differentiation. The increased plakoglobin, involucrin, and filaggrin expression and decreased vimentin levels suggested epithelial differentiation of hWJ-MSCs. However, it is appropriate to clarify that this cell behavior was cell donor dependent, and the synthesis of epithelial proteins was intracellular, which is probably attributed to the fact that hWJ-MSCs were not terminally differentiated [61]. Vimentin is a mesenchymal marker located at the intermediate filaments of the cytoskeleton and regulates the epithelial–mesenchymal transition process [62, 63]. Filaggrin and involucrin are important keratinization markers and play a critical role in skin barrier function [64], indicating early squamous epithelial formation [65]. Plakoglobin is a catenin family member and is a common component of both adherents junctions and desmosomes [66]; regulating intercellular junctions in keratinocytes. According to these results, the hADM scaffold and the air–liquid cell culture system enhance the differentiation of hWJ-MSCs toward the epithelial phenotype.

In addition to demonstrating the in vitro potential of hADM to promote adhesion, proliferation, the release of bioactive molecules, and transdifferentiation of hWJ-MSCs, we proceeded to evaluate the repair capacity of hADM and construct 1 in full-thickness excisional wounds using porcine biomodel, which cannot be repaired spontaneously [67]. The in vivo evaluation showed that hADM scaffold and construct 1 were grafted in the treated wounds and kept them hydrated. Histological analysis of the wounds treated with hADM and construct 1 showed that all skin layers were present, indicating a significant degree of re-epithelialization and formation of a basement membrane and parallel oriented collagen fibers, an indicator of adequate dermal organization. In addition, treatment with the dermal matrix significantly decreased wound contracture in the new tissue form compared to the control wound. However, hair follicles and sebaceous glands were not found in the repaired skin in any treatments. It should be noted that the treatments described here promoted closure of the lesion in only 30 days and significantly improved the appearance of the scar. Likewise, the absence of inflammatory cells confirmed the biocompatibility of hADM due to its low content of DNA and, in construct 1 by the hWJ-MSCs immunomodulatory properties [68].

Our results reported more significant signs of repair than those reported in a study on the same biomodel using decellularized fetal bovine skin substitute with autologous skin cell suspension [69], indicating that the wound closed in approximately 42 days after treatment and according to the histological result, the immature epithelium and presence of inflammatory cells in the wound treated with decellularized skin substitute were observed at 28 days. Likewise, the skin repair potential of construct 1 and hADM described here was higher than similar strategies evaluated in different animal models [67, 70, 71]. Although we did not use a positive control such as Integra™ seeded with keratinocytes in our in vivo assay, previous studies have shown that this alternative, evaluated in a porcine biomodel, fails to achieve epithelial stratification [72], and also produces hyperkeratinized scarring at week 8 of evaluation [73]. In contrast, our results showed that the hWJ-MSC/hADM construct or hADM scaffold promoted lesion closure at 30 days after implantation with complete epithelialization, thin and thick collagen fibers, non-hypertrophic scar, and contracture reduction, indicating a greater efficiency of skin repair compared to this dermal substitute. In addition, the biological properties of MSCs are superior to differentiated cells such as keratinocytes. Previous studies showed that production of immunomodulatory, proangiogenic and remodeling factors is greater in undifferentiated or multipotent cells than in mature cells like fibroblasts or keratinocytes [74]. Thus, the use of multipotent cells such as hWJ-MSCs in tissue constructs improves skin wound healing [74, 75]. On the other hand, a clinical trial about Integra efficacy in wound repair showed an induced higher foreign body reaction; however, this response is expected because it is produced by chemical crosslinking and contains bovine proteins and shark-derived glycosaminoglycan [76].

It is appropriate to communicate the main limitation to fabricating our construct is that it requires human cadaveric skin to obtain the hADM, and its procurement is highly dependent on the culture of donation. Besides, the dimensions of the construct for clinical use depend on the area of dermatome available commercially, which is 10 cm wide. On the other hand, the therapeutic application of the hADM/hWJ-MSCs constructs requires the development of clinical trials to define its safety and efficacy.

## Conclusion

Overall, we have generated a hADM scaffold following a practical and time-efficient protocol for skin decellularization that maintains the native structure of the ECM and considerably reduces the DNA content. Our hADM promoted cell adhesion and proliferation, making it an excellent scaffold for generating tissue constructs. We have demonstrated that constructs of hWJ-MSCs and hADM exposed or not to a proinflammatory stimulus can potentially produce growth factors related to wound repair, exceptionally high levels of HGF and bFGF, emphasizing that the release of paracrine factors and cell proliferation are highly desirable conditions for designing tissue engineering therapeutic products. Similarly, the epithelial differentiation results of hWJ-MSCs may indicate a significant contribution of the 3D architecture in natural dermis scaffolds to achieve differentiation of hWJ-MSCs toward non-mesodermal lineages such as epithelial lineage. In vivo testing confirmed the potential of both hADM and the hADM/ hWJ-MSC construct to repair full-thickness skin wounds, with significant improvement in the appearance of the newly formed tissue. These results suggest that hADM and the constructs obtained in this study represent a novel strategy for skin wound repair.

### Supplementary Information

**Additional file 1**. **Table S1**: Antibodies used in immunocytochemistry.

## Data Availability

The datasets used and/or analyzed during the current study are available from the corresponding author.

## Abbreviations

MSCs: Mesenchymal stromal cells
hWJ-MSCs: Human Wharton’s jelly mesenchymal stromal cells
hADM: Human acellular dermal matrix
hPL: Human platelet lysate
ECM: Extracellular matrix
IL-1α: Interleukin-1α
IL-1β: Interleukin-1β
IL-6: Interleukin-6
TNF-α: Tumor necrosis factor-α
EGF: Epidermal growth factor
bFGF: Basic fibroblast growth factor
HGF: Hepatocyte growth factor
PDGF: Platelet-derived growth factor
VEGF: Vascular endothelial growth factor
Ang I: Angiopoietin-I
HIV: Human immunodeficiency virus
HCV: Hepatitis C virus
HBV: Hepatitis B virus
HTLV: Human T-lymphotropic virus
PBS: Phosphate-buffered saline
PFA: Paraformaldehyde
DNA: Deoxyribonucleic acid
SEM: Scanning electron microscopy
TCP: Tissue culture plate
DMEM: Dulbecco’s Modified Eagle Medium-Thermo Fisher Scientific
