# Supplementary material for: Bioengineered skin constructs based on mesenchymal stromal cells and acellular dermal matrix exposed to inflammatory microenvironment releasing growth factors involved in skin repair
Source: Stem Cell Res Ther. 2023 Oct 26;14:306. doi: 10.1186/s13287-023-03535-w (PMC10601120; doi:10.1186/s13287-023-03535-w)
Supplement: Supplementary file 1 — Additional file 1. Table S1: Antibodies used in immunocytochemistry. [file 13287_2023_3535_MOESM1_ESM.pdf]

Supplementary table 1: Antibodies used in immunocytochemistry

Antibody	Reactivity	Concentration	Suppliers	Identifier
Primary antibodies				
Anti-Filaggrin	Human	2 µg/mL	Novus biologicals	Cat# NBP1-87528
Anti-Involucrin	Human	5 µg/mL	Biolegend	Cat#924401
Anti-JUP (Junction plakoglobin)	Human	10 µg/mL	Abnova	Cat# H00003728
Alexa Fluor® 594 anti-Vimentin	Human	2,5 µg/mL	Biolegend	Cat#677804
Secondary antibodies				
Alexa Fluor® 594 Goat Anti-Rabbit IgG	Rabbit	1:1000	Abcam	Cat#ab150080
Alexa Fluor® 594 Goat Anti-Mouse IgG	Mouse	1:1000	Abcam	Cat#ab150116
